# Reverse strategy to locally advanced breast implant-associated anaplastic large cell lymphoma: A case report

**DOI:** 10.3389/fonc.2022.1062389

**Published:** 2023-01-10

**Authors:** Sonia Cappelli, Francesco Marchesi, Marco Clementi, Letizia Perracchio, Francesca Palombi, Fabio Pelle, Claudio Botti, Maurizio Costantini

**Affiliations:** ^1^ Department of Surgery, Division of Breast Surgery, Istituti di Ricovero e Cura a Carattere Scientifico (IRCCS) Regina Elena National Cancer Institute, Rome, Italy; ^2^ Hematology Unit, Department of Research and Clinical Oncology, Istituti di Ricovero e Cura a Carattere Scientifico (IRCCS) Regina Elena National Cancer Institute, Rome, Italy; ^3^ Department of Applied Clinical Sciences and Biotechnology, University of L’Aquila, L’Aquila, Italy; ^4^ Pathology Department, Istituti di Ricovero e Cura a Carattere Scientifico (IRCCS) Regina Elena National Cancer Institute, Rome, Italy; ^5^ Department of Surgery, Division of Plastic and Reconstructive Surgery, Istituti di Ricovero e Cura a Carattere Scientifico (IRCCS) Regina Elena National Cancer Institute, Rome, Italy

**Keywords:** breast implant-associated anaplastic large cell lymphoma (BIA-ALCL), reverse-strategy, neoadjuvant chemotherapy, stem cell transplant, conservative surgery

## Abstract

Breast implant-associated anaplastic large cell lymphoma (BIA-ALCL) is a rare T-cell lymphoma associated with textured breast implants. The most common presentation is a periprosthetic seroma that occurs at least 1 year after an aesthetic or reconstructive implantation, and in these cases, the surgical treatment seems to be successful. More rarely, BIA-ALCL presents with locally advanced mass-formed disease and a related regional lymph node involvement. In all these cases with worse prognosis, a multidisciplinary approach is required, including adjuvant chemotherapy, radiation therapy, and surgery. We present a clinical case of a 49-year-old woman who developed on the left side of the breast a mass-formed stage 3 BIA-ALCL 15 years after a bilateral breast augmentation with textured silicone implant. Our multidisciplinary team (MDT) scheduled the patient for a “reverse-strategy” sequential approach consisting of induction chemotherapy, hematopoietic stem cell mobilization, and harvest followed by autologous stem cell transplant (ASCT). After 100 days from the stem cell transplant, the patient showed a complete pathologic response and was a candidate for radical surgery. She underwent removal of both implants with total en bloc capsulectomy. On the left site, the periprosthetic mass was also en bloc removed. We did not perform any axillary dissection. Our surgical and hemato-oncological teams followed the patient every 3 months, and no local or systemic recurrences were observed 24 months after surgery. This case report has demonstrated the effectiveness of neoadjuvant chemotherapy as part of a “reverse strategy” in selected cases of advanced-stage BIA-ALCL in which it was not possible to perform an immediate radical surgery. Furthermore, in our case, the de-escalation strategy adopted permitted a less demolitic surgery with good functional and aesthetic results.

## Introduction

Breast implant-associated anaplastic large cell lymphoma (BIA-ALCL) is a recently recognized non-Hodgkin lymphoma of T-cell origin. Despite its low incidence, the increasing use of breast implants for aesthetic reconstruction or post-mastectomy purposes poses BIA-ALCL as an emerging medical challenge (1). All clinical case reports have demonstrated a strong relationship between BIA-ALCL and textured breast implants.

Although most patients with BI-ALCL have a relatively indolent clinical course presenting as a delayed effusion or persistent seroma around the implants, one-third of the cases develop a peri-capsular mass and a small number of lymph-node involvement or distant disease (2, 3).

The National Comprehensive Cancer Network (NCCN) guidelines and the UK guidelines on the Diagnosis and Treatment of BIA-ALCL suggest that during the early stage, surgical removal of the implant with the surrounding capsule intact usually has a curative effect. For mass-forming cases, surgery was also indicated with a total en bloc capsulectomy with removal of any associated mass and abnormal regional lymph nodes followed by adjuvant chemotherapy and/or radiotherapy (4, 5). In advanced-stage patients, the role of neoadjuvant therapy has not been investigated yet.

We report a case of a woman with locally advanced BIA-ALCL treated with an unconventional combined approach in which a sequential chemotherapeutic program was applied in a neoadjuvant setting (reverse strategy).

## Case report

**Figure 1:**
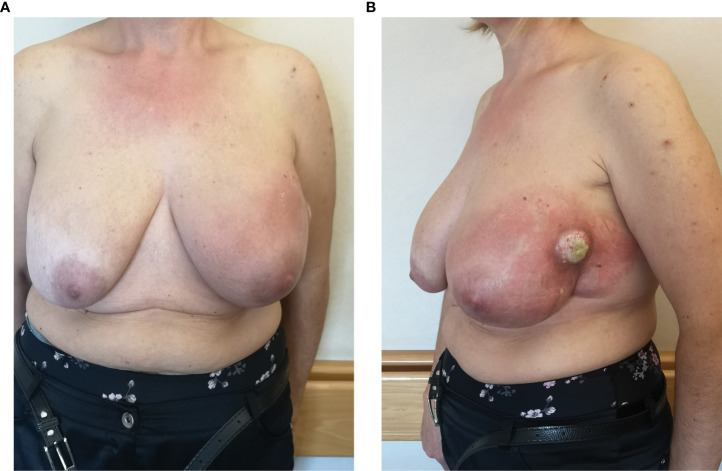
Clinical presentation at the recruitment: frontal view **(A)** and lateral view **(B)**.

A 49-year-old woman with a history of bilateral breast augmentation with silicone implant macro textured 15 years earlier presented with the appearance of a palpable large mass associated with a volumetric increase of the left breast. She had no postoperative complications or previous trauma to the breast, nor did she undergo any additional breast surgery before presentation to the clinic. The patient did not show any significant previous clinical history or relevant comorbidities. Clinical examination revealed swelling, erythema, palpable mass, and *peau d’orange* on the left breast (**Figures 1A, B**). The patient underwent breast ultrasound and magnetic resonance imaging. The latter showed signs of intracapsular ruptures.

**Figure 2:**
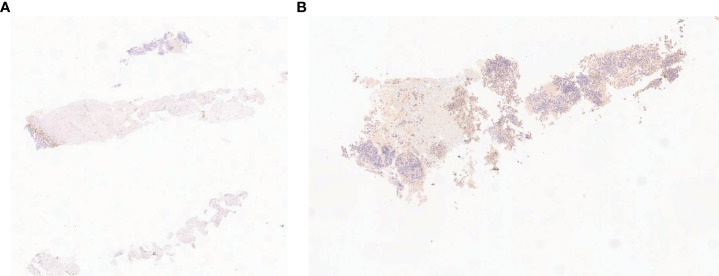
Fine needle cytology: neoplastic proliferation of large round cells with pleomorphic nuclei, negative for epithelial marker CK **(A)** and positive for linfoid marker and vimentin **(B)**.

On the same side, it had morbid tissue with a necrotic component localized in the external quadrant extending from the skin to the muscle layer (4 × 8 cm); the local swelling and the increase in skin thickness affect all the breast tissue. In addition, suspicious axillary lymph nodes have been reported in the same side. Given the complexity of the case, the patient was sent to our Breast Surgery Department of the “Regina Elena National Cancer Institute” in Rome, considered as a reference point in central-southern Italy for breast cancer (6, 7). We subjected the patient to 18-FDG positron emission tomography/computed tomography scan (PET/TC), which showed a focal increase of the left breast tissue intake (SUV max 39.4) going from the skin to the muscular layer of the outer quadrants and of the axillary and retro-pectoral lymph nodes (SUV max 7.8; dmt max 26 mm). Clinical and radiographic evaluations of the right breast did not show any abnormalities. The patient was subjected to fine needle aspiration of mass; cytology exam showed round, large cells with pleomorphic and cerebroid nuclei (CD45+++, Vimentin++, Keratin−). The following percutaneous biopsy of the mass demonstrated a proliferation of phenotypically aberrant population of large cells that expressed CD30 and CD45, negative for CD3, CD20, and ALK1, diagnostic for anaplastic large cell lymphoma, ALK-negative (ALK-ALCL) (**Figures 2A, B**) (8).

**Figure 3:**
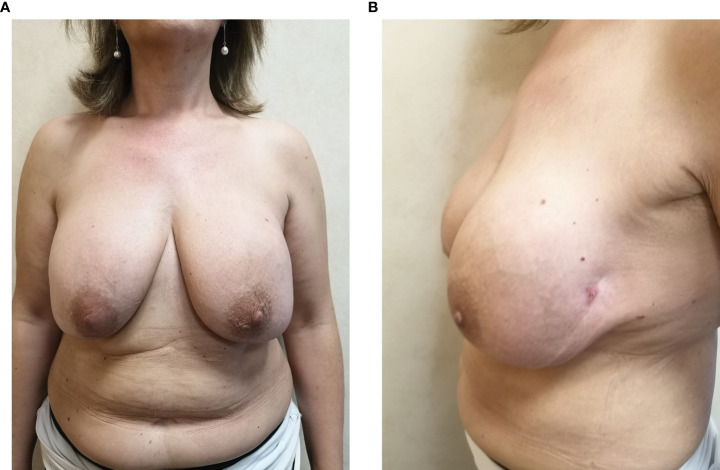
Clinical response after chemotherapy: frontal view **(A)** and lateral view **(B)**.

Following a multi-disciplinary tumor board discussion, involving hematologists, surgeons, pathologists, and radiotherapists, an immediate surgical treatment was considered not indicated, as a consequence of the extremely advanced disease stage not allowing a minimally invasive surgical intervention. After the administration of informed consent, we decided first to start a sequential chemotherapeutic approach consisting of induction chemotherapy, hematopoietic stem cell mobilization, and harvest followed by autologous stem cell transplant (ASCT), according to our institutional policy and international guidelines about treatment of advanced-stage ALK-negative peripheral T-cell lymphoid malignancies (9–12). In particular, induction chemotherapy consisted of six courses of alternating CHOEP (cyclophosphamide, vincristine, doxorubicin, etoposide, and prednisone; three cycles) and DHAP (cisplatin, cytarabin, and dexamethasone; three cycles). Hematopoietic stem cells were collected after the last DHAP cycle (CD34+ × 10^6^/kg collected: 7.024). At the end of the induction phase, a disease assessment performed by a CT/PET scan showed a complete remission. Afterwards, a consolidation treatment with ASCT after BEAM conditioning regimen (BCNU, etoposide, cytarabine, and melphalan) was finally performed (**Figures 3A, B**).

**Figure 4:**
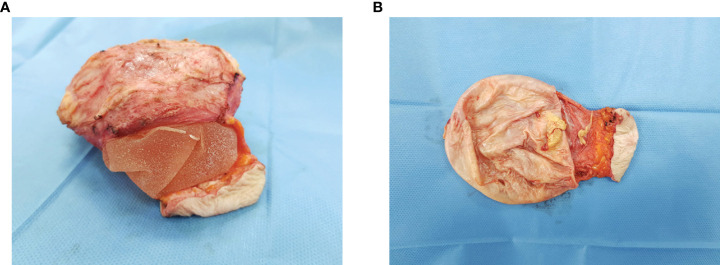
Surgical specimen: implants with total en bloc capsulectomy **(A)** and periprosthetic tissue previously involved by BIA-ALCL **(B)**.

One hundred days after the stem cell transplant, she underwent removal of both implants with total en bloc capsulectomy. On the left, the site of the periprosthetic mass was also removed (**Figures 4A, B**).

**Figure 5:**
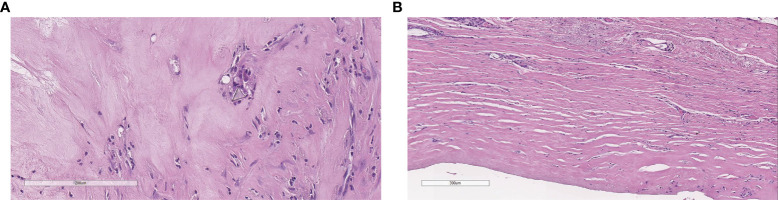
Complete pathological response: histology does not show neoplastic cells with a polyurethane crystal and rare inflammatory cells **(A)**, flat synovial metaplasia, and foreign body giant cell granulomas **(B)**.

We did not perform any axillary dissection. The histology, performed as reported by Lyapichev et al. (13), revealed a complete pathologic response with CD30 immunohistochemistry negative (**Figures 5A, B**).

Given his complete pathological response, multidisciplinary consensus did not consider adjuvant radiotherapy necessary. The patient was followed by our surgical and hemato-oncological teams every 3 months and no local or systemic recurrences were observed 24 months after surgery.

## Discussion

BIA-ALCL has been identified as an emerging disease entity and represents a novel variant of the clinic pathologic subtypes of anaplastic large cell lymphoma (ALCL) associated with breast implants with a textured outer. Since its first description in 1997 by Keech and Creech (1, 14), an increasing number of cases have been reported in literature (15), and recently, the World Health Organization has listed BIA-ALCL as a unique pathological entity (16).

The most common presentation of BIA-ALCL is a large spontaneous periprosthetic fluid collection occurring at least 1 year and on the average from 7 to 10 years following the implantation with a textured surface breast implant. In addition to large fluid collections and delayed seromas, 8% to 24% of patients present an associated palpable mass and 4% to 12% present with lymphadenopathy (4). Less than 5% of the cases are unfrequently described and manifest local and systemic signs, such as rash, fever, and capsular contracture (17).

Surgical excision is the established standard of care to treat the localized disease, but the BIA-ALCL treatment algorithm suggested by international guidelines is limited in case of unusual, progressive, or complex patient scenarios (18, 19). This smaller subset of patients that present with a tumor mass associated with the fibrous capsule or lymph node involvement are more likely to have worse prognosis. Miranda et al. found that only 72% of patients with tumor masses achieved remission, as opposed to 93% when disease was confined to the fibrous capsule (20). Ferrufino-Schmidt et al. found out a significant reduction in 5-year overall survival in patients with lymph node involvement compared to patients who are not clinically or radiologically suspected to have lymph node disease (75% vs. 97.8%) (21).

Our clinical case falls into one of those advanced BIA-ALCL stages (stage III) (13, 22) with worse prognosis. The patient had a large mass forming on the left side extending from the skin to the muscle layer and suspected ipsilateral axillary lymph nodes.

Our multidisciplinary oncology board, which involved hematologists, surgeons, pathologists, and radiotherapists, did not consider the patient as a candidate for immediate surgery due to the advanced stage of the disease. Such an intervention would have been extremely destructive and not safe. Keeping in mind the importance of obtaining safe negative surgical margins and conservative surgery with oncoplastic techniques, the patient was scheduled to administer chemotherapy and ASCT before surgery, reversing the commonly recommended strategy (reverse strategy).

In accordance with our institutional policy and international guidelines about treatment of advanced-stage ALK-negative peripheral T-cell lymphoid malignancies (9–12), patients were submitted to induction chemotherapy by six courses of alternating CHOEP and DHAP. Finally, a consolidation treatment with ASCT after BEAM conditioning regimen was performed. The chemotherapeutic regimen used in our patients is in line with the regimen proposed in literature to treat advanced BIA-ALCL in an adjuvant setting (4, 5). At the end of the induction phase, a clinical and radiological restaging manifested a complete remission of the disease.

Keeping in mind the pivotal role of “free” surgical margins to reduce local recurrences (22), the surgical procedure performed on treated tissue allows for a less destructive surgery without compromising its radical nature. In our case, a radiological complete remission was obtained, 100 days from the stem cell transplant; the patient underwent bilateral capsulectomy with, on the left side, a local excision of previous pericapsular mass to ensure that no tumor was present at the margins.

Contrary to guideline recommendations, we did not perform any axillary dissection, despite the initial suspicion of multi-lymphonodal involvement. A complete response was obtained for the concomitant axillary lymph node, as expected after the medical management of common ALCL so that we avoided needless axillary dissection. Our patient was not interested in maintaining an enlarged breast and asked to avoid an immediate reconstruction with smooth implants. At follow-up, a device-free reconstruction was planned, leaving the breast in a natural-looking shape. Two other cases, in which neoadjuvant therapy has been administered to treat advanced BIA-ALCL, have been reported in the literature (18, 19).

In both cases, a complete pathological response was achieved after neoadjuvant chemotherapy, and the adjuvant radiotherapy was deemed unnecessary, following a multidisciplinary consensus.

Our case has shown that the use of neoadjuvant chemotherapy is effective in cases of advanced BIA-ALCL, when immediate surgical treatment is not indicated for the extension of the disease. In these scenarios, the reverse strategy, which involves the use of neoadjuvant chemotherapy, may obtain near-clinical complete response allowing for conservative surgery.

The incidence of BIA-ALCL has continued to rise, but consistent data regarding recommendations for patients with a more aggressive presentation of BIA-ALCL are still lacking in the literature. In this optic, the role of unconventional therapeutic strategy should be investigated. Our report underlines the important role of neoadjuvant chemotherapy in BIA-ALCL to control disease burden before tumor resection in selected cases in which radical surgery is not possible or precluded due to the general condition of the patient.

## Consent to participate

Informed consent was obtained from the participant for the publication of this case report including all data and images.

## Data availability statement

The raw data supporting the conclusions of this article will be made available by the authors, without undue reservation.

## Ethics statement

The studies involving human participants were reviewed and approved by Central Ethics Committee IRCCS Lazio – IRCCS I.F.O. with number RS1719/22. The patients/participants provided their written informed consent to participate in this study. Written informed consent was obtained from the individual(s) for the publication of any potentially identifiable images or data included in this article.

## Author contributions

SC and FM wrote the main manuscript, its acquisition data, and the interpretation. LP, FPa, and FPe contributed to data acquisition. MCI made a critical review of the article. CB and MCo were in charge of the final approval of the version to be published. The final manuscript has been read and approved by all the authors.
